# Shear bond strength of resin bonded zirconia and lithium disilicate – effect of surface treatment of ceramics and dentin

**DOI:** 10.1080/26415275.2022.2038177

**Published:** 2022-02-16

**Authors:** Mina Aker Sagen, Linda Vos, Jon E. Dahl, Hans J. Rønold

**Affiliations:** a Nordic Institute of Dental Materials (NIOM), Oslo, Norway; b Department of Prosthetic Dentistry, Institute of Clinical Dentistry, Faculty of Dentistry, University of Oslo, Oslo, Norway

**Keywords:** Ceramics, surface modification, resin cement, dentin, surface roughness

## Abstract

**Objectives:**The purpose of the study was to investigate the effect of ceramic surface pretreatment, effect of resin cement and dentin surface roughness on shear bond strength.

**Methodology:** Zirconia rods (*n* = 140) were randomly assigned to air born particle abrasion with aluminum oxide (Al_2_O_3_) or hot etching with potassium hydrogen difluoride (KHF_2_). Lithium disilicate rods (LDS; *n* = 50) etched with hydrofluoric acid served as reference material. In Part 1 of the study, ceramic rods were cemented to bovine dentin using 5 dual-polymerizing resin cements (Variolink Esthetic, Multilink Automix (Ivoclar Vivadent), Duo-Link (BISCO Dental), Panavia F2.0 (Kuraray Dental), RelyX Unicem (3 M)). Shear bond strength was tested and fracture morphology determined. In Part 2 of the study, test groups with the highest frequency of adhesive fractures between cement and dentin were selected for further bond strength testing with different surface roughness of dentin; ground with P1200 or P80 silicon carbide paper. Dentin samples were fractured vertically to the cemented surface and the adherence between cement and dentin was studied.

**Results:** The results of Part 1 showed that hot etching of zirconia significantly improved bond strength to Duo-Link cement. In Part 2, RelyX Unicem showed significantly higher bond strength to P1200 compared to P80 ground dentin. For Variolink Esthetic, bond strengths to P1200 and P80 ground dentin were similar. Adhesive fracture between cement and dentin dominated.

**Conclusions:** A smooth dentin surface (P1200) improved bond strength to RelyX Unicem. Surface roughness was not important for Variolink Esthetic.

## Introduction

Debonding of ceramic restorations is a technical complication reported in many studies [1–4] and might occur either between cement and tooth substance, between cement and ceramic or within the cement layer.

Surface treatment of zirconia aims at increasing bond strength of resin cemented restorations. Still there is no consensus about the optimal treatment [5]. Hot etching of the zirconia surface using potassium hydrogen difluoride (KHF_2_) presented by Ruyter et al. [6] showed promising bond strength when compared to the more traditional aluminum oxide (Al_2_O_3_) particle abrasion. After hot etching the surface is left with a roughness that promotes micro-mechanical retention. The melting of KHF_2_ also leaves the zirconia surface fluoridated, and after steam cleaning and ultrasonically cleaning, active hydroxyl sites are available for establishing chemical bonds to bonding agents. In addition, a higher amount of the tetragonal crystal phase was observed [6], indicating that the hot etching method leads to less material damage. Similar results were recently published [7]. In Sagen et al. [8] KHF_2_ etched and Al_2_O_3_ particle abraded zirconia rods were cemented to dentin using resin cements and tested for tensile bond strength. The results showed that the bond strength was similar for the two surface treatments for four out of five cements.

Dentin topography and composition influence the bond to resin cement [9,10]. Surface roughness affects the area available for contact between cement and dentin and for mechanical interlocking [9,11]. Preparation of tooth substance for indirect restorations performed with different bur grit affects the retention of the final restoration [9,12,13]. However, there is no consensus about which grit the preparation diamond should have [12].

Dual-polymerizing resin cements can be divided into different types according to their adhesive mechanism; Etch-and-rinse, self-etching or self-adhesive [14–16]. Three-step etch-and-rinse adhesives have been the golden standard adhesive for decades [17], but the many steps involved makes these adhesives time-consuming and disposed to error. Comparative studies have shown that cements with less time-consuming protocols perform just as good or even better in bond strength testing [8,16]. The aim of this study was to evaluate the effect of both ceramic and dentin surface modifications on bond strength of cemented test specimens. For dentin, the different surface roughnesses tested was put in relation to the type of cement in order to identify the optimal combination for bond strength.

The following hypotheses were tested: 1) no effect of different surface treatments of ceramic on shear bond strength, 2) no effect of different resin cements on shear bond strength and 3) no effect of dentin surface roughness on shear bond strength of KHF_2_ etched zirconia.

## Materials and methods

### Preparation of specimens

Preparation of specimens was performed according to protocol published in Sagen et al. [8]

Bovine mandibles (4–6 years old) were obtained from the Norwegian food producer Nortura. A total of 190 bovine mandibular incisors were extracted, cut and mounted in epoxy resin (EpoFix, Struers, Copenhagen, Denmark) with buccal surface exposed.

Circular zirconia (*n* = 100, Starceram Z, H.C. Starck Ceramics GmbH, Selb, Germany) and lithium disilicate (*n* = 50, IPS e.max CAD, Ivoclar Vivadent, Schaan, Liechtenstein, ) rods (5 mm diameter, 11.5 mm length) were produced by CAD/CAM technique (Solidworks CAD, Dassault Systemes, Waltham, Massachusetts. hyperDENT CAM software, FOLLOW-ME!, Munich, Germany. Milled in Röders RXD5C, Soltau, Germany). The rods were ground at one end using P500 silicon carbide paper (SiC, Struers, Copenhagen, Denmark) to obtain a uniform surface roughness. Before further surface treatment the rods were cleaned with a dental steamer (Steamer X3, Amann Girrbach, Pforzheim, Germany) and thoroughly air-dried.

### Surface treatment of zirconia and lithium disilicate rods

Surface treatment of ceramic rods was performed according to Sagen et al. [8].

Zirconia rods (*n* = 100) were randomly distributed to two different surface treatment groups:

1. Zir A: air borne particle abrasion with 50 µm Al_2_O_3_ at a 10 mm distance perpendicular to the surface at 2.5 bar for 10 s. The rod was placed in a holder that had a rotational movement.
2. Zir E: hot etching with KHF_2_ powder at 280 °C for 10 min. The fine powder was applied to the cementation surface and melted when heated in a furnace (Jelenko, acc-therm II 2000, Armonk, NY).

Additional zirconia rods (*n = 40,* Dental Direkt Bio ZW iso, Spenge, Germany) were hot etched by KHF_2_.

After surface treatment rods were steam cleaned, ultrasonically cleaned in distilled water for 15 min and air dried.

Lithium disilicate rods (LDS) served as reference material and were etched with 4.5% HF acid (IPS Ceramic Etching Gel, Ivoclar Vivadent, Schaan, Liechtenstein) for 20 s, thoroughly cleaned in running water and air dried.

Selected rods were studied in scanning electron microscope (SEM, Hitachi Analytical TableTop Microscope/Benchtop SEM TM3030, Tokyo, Japan) to visualize the morphology after surface treatment.

### Dentin surface preparation and evaluation

Bovine mandibular incisors embedded in epoxy resin were randomly distributed to three surface preparation groups. In Part 1 of the bond strength study the dentin surfaces (*n* = 150) were ground with P500 SiC paper (Struers, Copenhagen, Denmark) on a horizontal grinding machine and cleaned by pumice powder dispensed in water. In Part 2 of the bond strength study the dentin surfaces were ground using P80 and P1200 (*n* = 2*20) SiC papers, respectively.

All specimens were ground until > 5 × 5 mm dentin surface was exposed. Grinding was performed under running water and the teeth were kept in distilled water until cementation.

Samples of bovine dentin ground with P80 and P1200 were studied in SEM (Tabletop Microscope, HITACHI, TM4000Plus, Tokyo, Japan) and 3D surface topography and arithmetical mean height (Sa) was assessed (Hitachi map 3D Standard 7.4, Tokyo, Japan).

### Cementation

Cementation was performed according to the manufacturers’ instruction for use and primers were applied when recommended (Table 1). In addition, a standardized 8.7 N seating load was applied using a cementation jig. The cements were light cured for 20 s from four directions. Based on a previous study on cement layer thickness in similar test specimens [18], the average thickness was estimated to be 20–40 µm.

**Table 1.** Cements used and their adhesive methods to ceramic and tooth substance.

Cement	Pretreatment of ceramic	Pretreatment of tooth substance
Variolink Esthetic DC (Ivoclar Vivadent, Schaan, Liechtenstein)	Monobond Plus^a^	Two-step etch-and-rinse: Phosphoric acid etchant and Adhese Universal
Multilink Automix (Ivoclar Vivadent, Schaan, Liechtenstein)	Monobond Plus^a^	One-step self-etching: Multilink Primer A and B
Duo-Link (BISCO Dental, Schaumburg, IL)	Z-Prime Plus^b^, Bis-Silane Parts A&B, D/E Resin^c^	Three-step etch-and-rinse: Phosphoric acid etchant, All-Bond 2 primer A and B, Pre-Bond Resin
Panavia F 2.0 (Kuraray Noritake Dental Inc., Tokyo, Japan)	Clearfil Ceramic Primer Plus^a^	One-step self-etching: ED primer 2 liquid A and B
RelyX Unicem (3M, Maplewood, MN)	No pretreatment of zirconia, Bis-Silane Parts A&B^c^	Self-adhesive: No pretreatment

^a^Universal primer for both zirconia and glass ceramics; ^b^selective zirconia primer; ^c^selective pre-treatment of glass ceramics.

After cementation the test specimens were kept in 37 °C distilled water for 24 h, and thereafter thermocycled 5000 cycles in 5 and 55 °C water baths before shear bond strength testing.

### Shear bond strength testing

A universal mechanical testing machine (Lloyd LRX, Lloyd Instruments Ltd, Leichester, UK) was used to apply shear force (1.00 mm/min cross-head speed) to the interface of the test specimen. The cross-head was placed close to the dentin surface. Force at break (*N*) and shear bond strength (MPa) were registered in Nexygen DF Force Measuring Software (AMETEK, Inc., Berwyn, PA).

### Part 1: ceramic surface and bond strength

For dentin ground with P500 SiC paper, ten rods of each of the three ceramics were cemented with the same five dual-polymerizing resin cements as used in Sagen et al. [8] (Table 1) and submitted to shear bond strength testing and fracture characterization. Both dentin surfaces and ceramic rods were studied in light microscopy (American Optical Stereo Star/Zoom, model 570, American Optical Corporation, Buffalo, NY). Five different types of fracture morphologies were registered: 1) adhesive between cement and dentin; 2) adhesive between cement and ceramic; 3) cohesive in cement; 4) cohesive in dentin; 5) combination of adhesive and cohesive fractures.

### Part 2: dentin surface and bond strength

Test groups with the highest frequency of adhesive fractures between cement and dentin were selected for evaluating the effect of dentin surface roughness on bond strength. Ten KHF_2_ etched zirconia rods were cemented with each of the cements Variolink Esthetic and RelyX Unicem to dentin ground with P80 (rough) and P1200 (smooth) grit SiC paper. After shear bond strength testing, liquid nitrogen was used for fracturing selected samples of bovine dentin vertically to the cemented area. Fractured samples were studied and photographed in SEM.

### Statistical analysis

Statistical analysis was performed using the STATA SE version 16.1 (StataCorp LLC, College Station, TX), and R version 4.0.3 (R, Vienna, Austria) . Comparisons of mean bond strength were performed using Student’s t-test with significance level < .05. Pictures were made using *ggplot2*-package in R.

## Results

### Part 1: ceramic surface and bond strength

**Figure 1.**
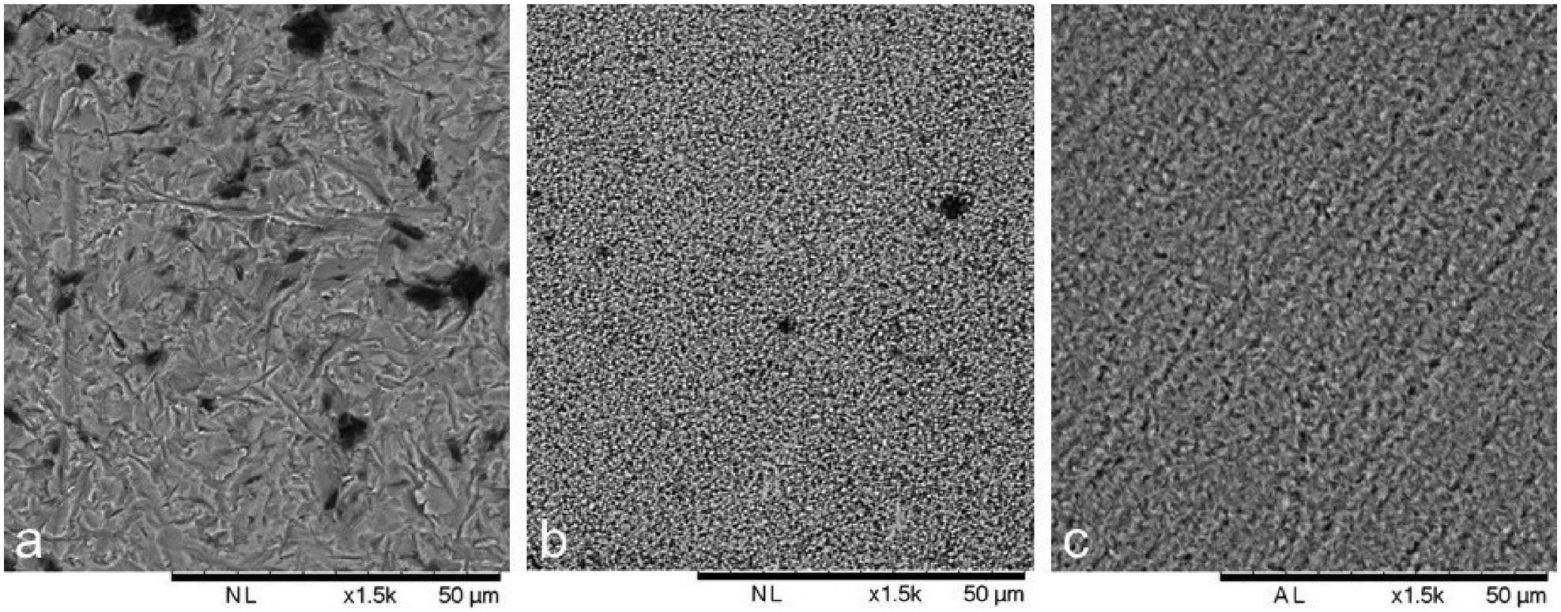
SEM images of air borne particle abraded zirconia (a), KHF_2_ etched zirconia (b) and hydrofluoric acid etched lithium disilicate (c). Bar represents 50 µm.

Morphology after the different surface treatments is visualized in Figure 1. As reported in a previous study [8] the Sa-value of Zir E was 0.12–0.13 µm, which was statistically significantly lower than both Zir A (Sa-value: 0.53–0.59 µm) and LDS (Sa-value: 0.18–0.25 µm).

**Figure 2.**
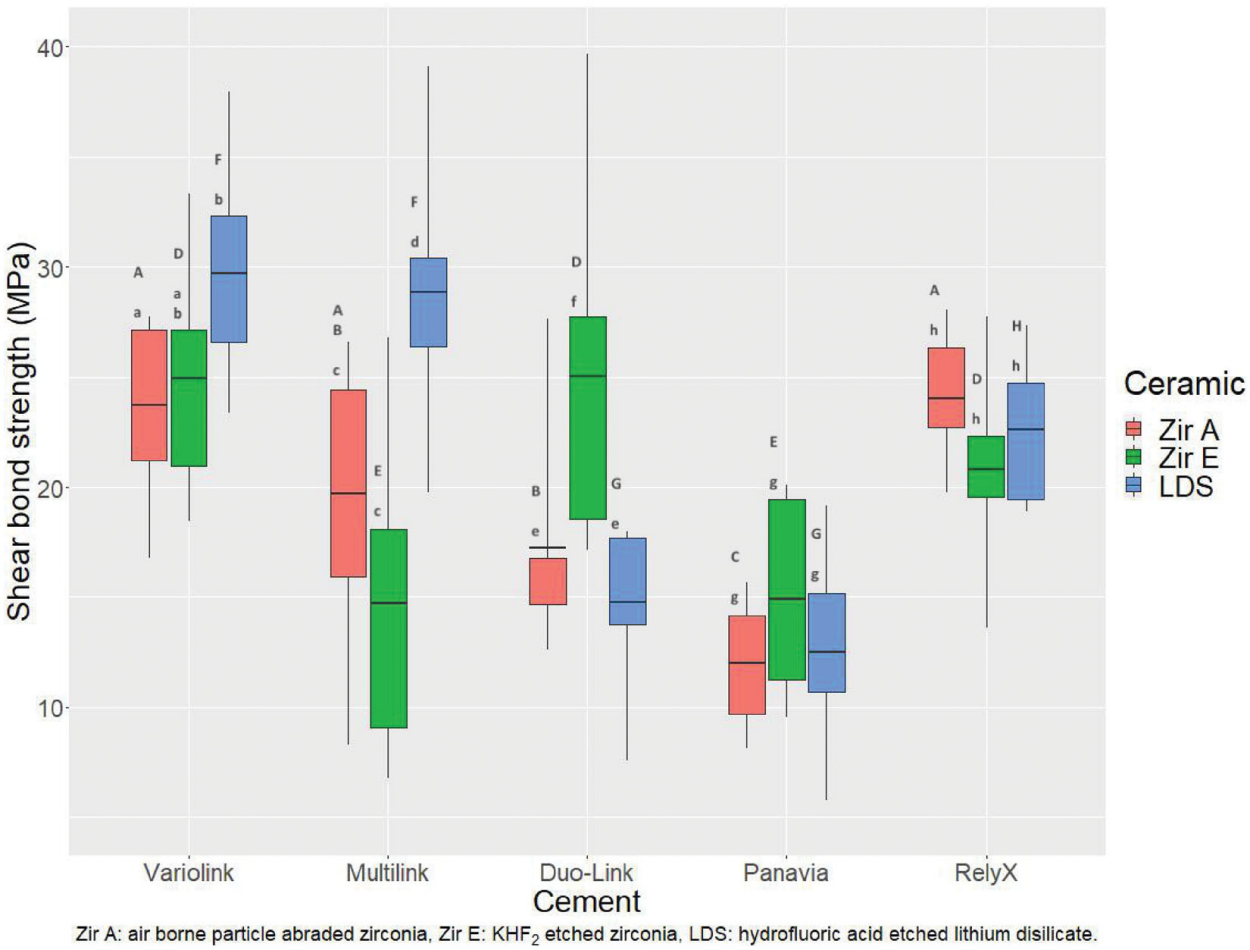
Box plot of shear bond strength of ceramic rods cemented to dentin (P500). The horizontal line represents the mean value, lower part of the box represents 25% quartile, the upper part of the box represents 75% quartile. The vertical lines represent a 90% confidence interval. Different lowercase letters illustrate significant difference (*p* < .05) between Zir A, Zir E and LDS for each cement. Different uppercase letters illustrate significant differences (*p* < .05) between cements for each ceramic.

There was no significant difference in bond strength between Zir A and Zir E for all cements used except for Duo-Link which promoted a higher bond strength of Zir E (Figure 2). The bond strength for the reference group (LDS) was higher, similar or lower than the zirconia groups.

Variolink Esthetic, Multilink Automix and RelyX Unicem resulted in the significantly highest bond strength for Zir A, whereas for Zir E Variolink Esthetic, Duo-Link and RelyX Unicem promoted the highest bond strength. For LDS, Variolink and Multilink resulted in significantly higher bond strength than the other cements (Figure 2).

The highest mean shear bond strength was observed for LDS cemented to dentin using Variolink Esthetic and the lowest for Zir A cemented to dentin using Panavia F2.0.

**Figure 3.**
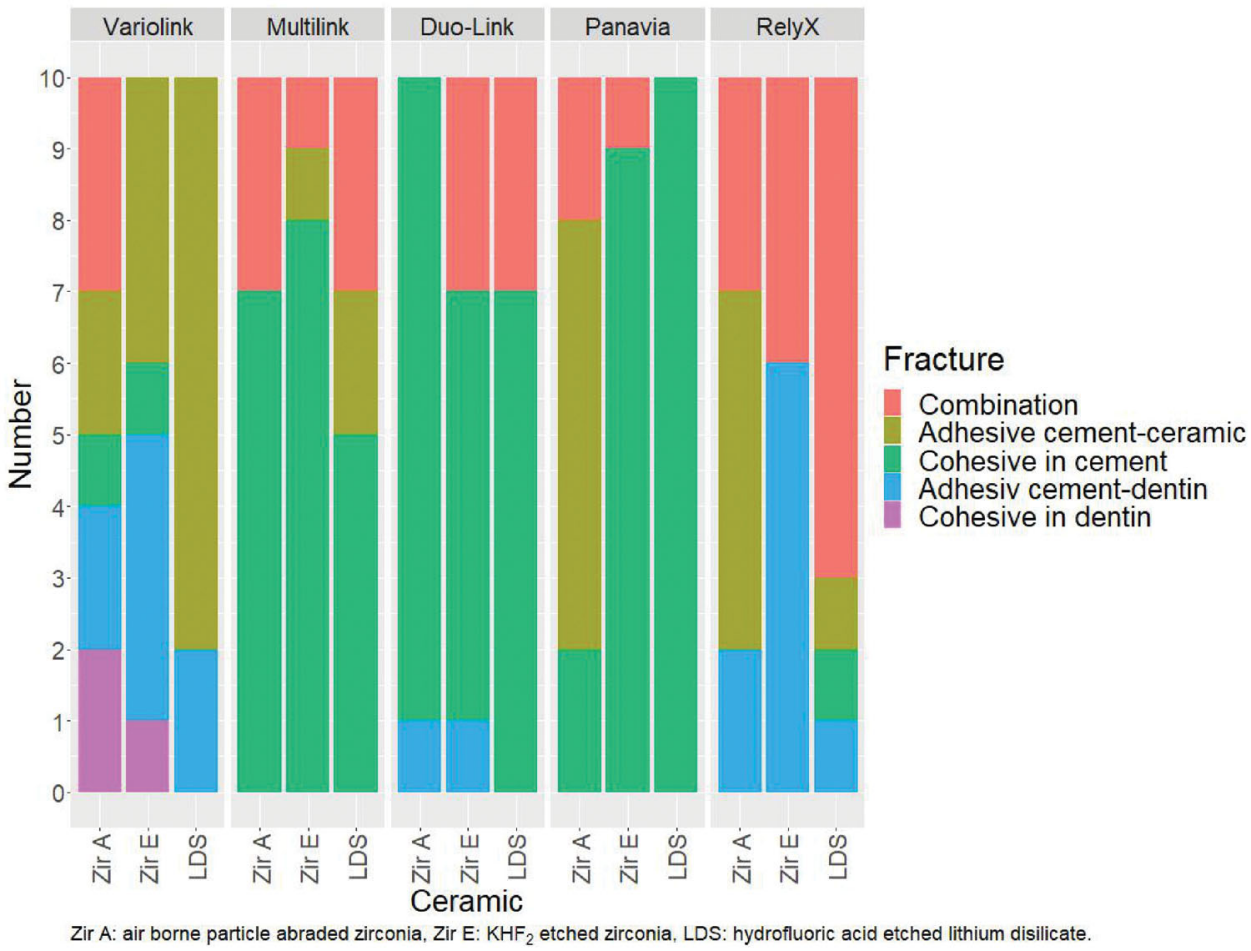
Fracture morphology after shear bond strength testing for ceramic rods cemented to dentin ground with P500 SiC paper.

Cohesive fracture in cement and combined adhesive and cohesive fractures dominated when Multilink Automix, Duo-Link and Panavia F2.0 were used for cementing ceramic rods to dentin ground with P500 SiC paper. The highest frequency of adhesive fracture between cement and dentin was observed when Variolink Esthetic and RelyX Unicem were used for cementing Zir E (Figure 3).

### Part 2: dentin surface and bond strength

**Figure 4.**
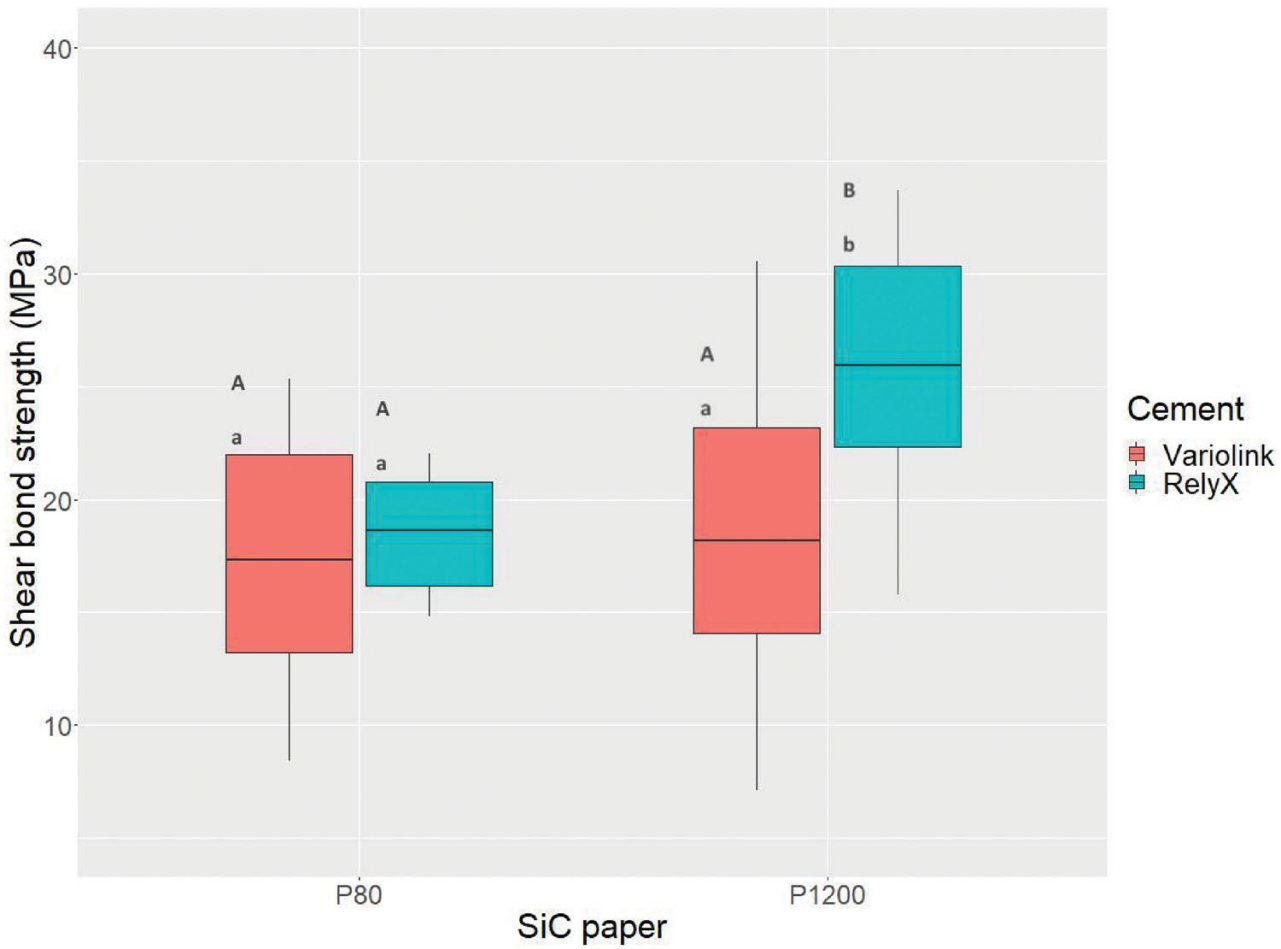
Box plot of shear bond strength for Zir E cemented to dentin ground with P80 and P1200 grit SiC paper. The horizontal line represents the mean value, lower part of the box represents 25% quartile, the upper part of the box represents 75% quartile. The vertical lines represent a 90% confidence interval. Different lowercase letters illustrate significant differences (*p* < .05) between cements for each surface roughness. Different uppercase letters illustrate significant differences (*p* < .05) between P80 and P1200 for each cement.

For dentin ground with P80 (Sa-value: 5.40 µm) and P1200 (Sa-value: 0.50 µm) SiC papers, the highest mean shear bond strength was observed when RelyX Unicem was used for cementing to P1200 dentin (Figure 4). Thus, P1200 resulted in significantly higher bond strength compared to P80 for RelyX Unicem, but for Variolink Esthetic no difference was observed between the two surfaces. Concerning the cements, RelyX Unicem resulted in significantly higher bond strength compared to Variolink Esthetic when dentin was ground with P1200, but for P80 no difference was observed (Figure 4).

**Figure 5.**
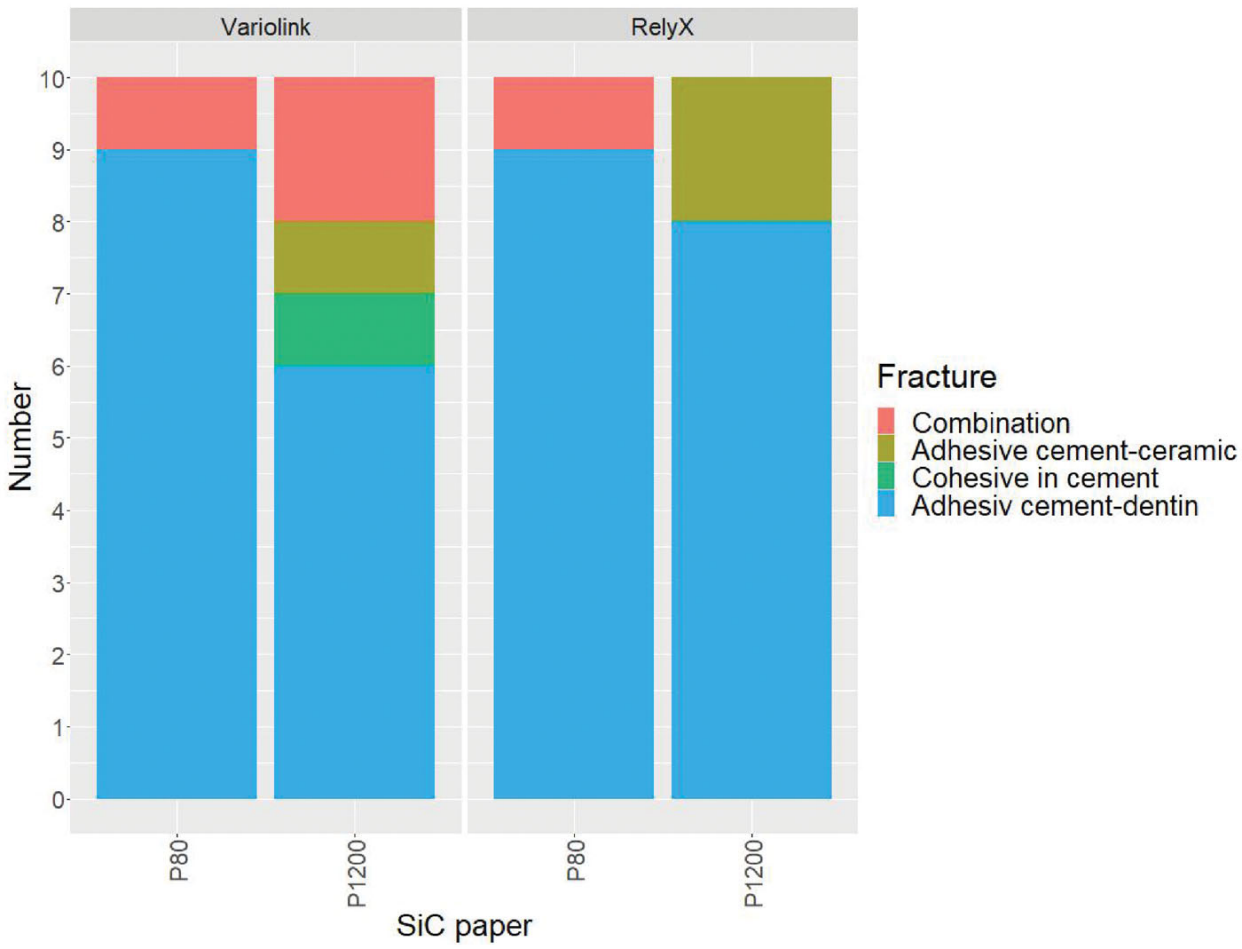
Fracture morphology after shear bond strength testing of Zir E cemented to dentin ground with P80 and P1200 SiC paper.

**Figure 6.**
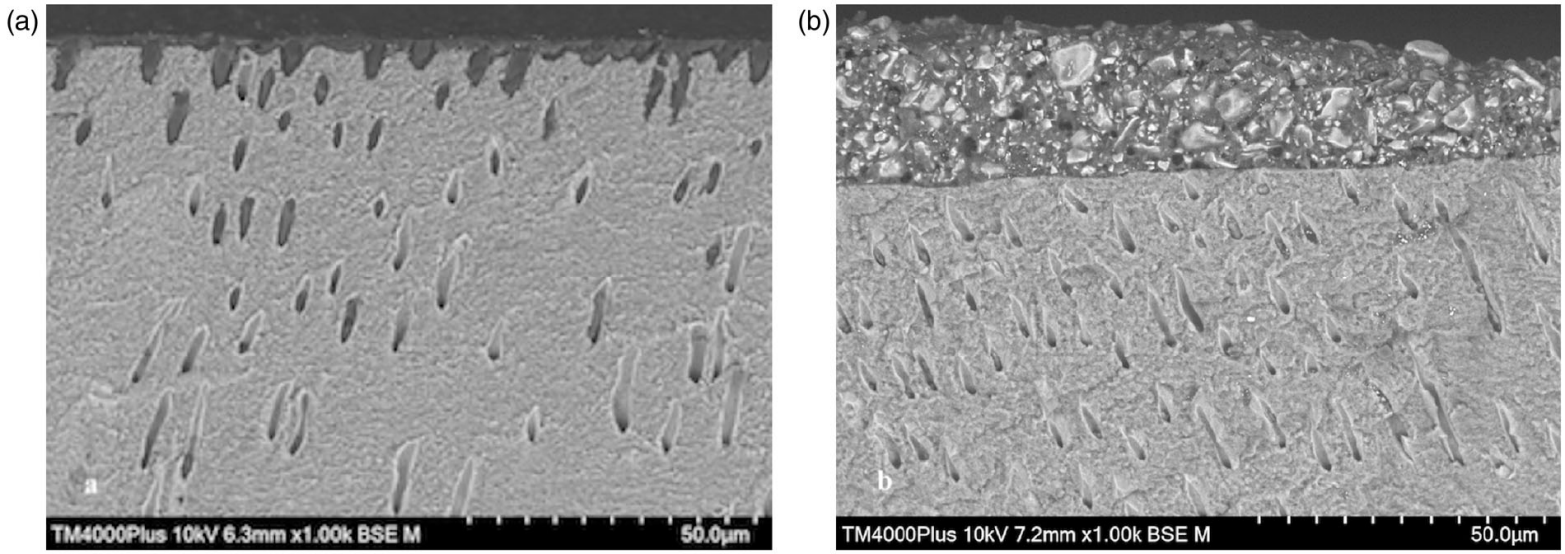
SEM images of dentin and remnants of cement after shear bond strength testing of Zir E cemented with Variolink (a) and RelyX (b) to dentin ground with P1200 SiC paper. 6a: Resin tags in dentin tubules. 6b: Remnant of RelyX on the dentin surface showing that the cement did not penetrate the dentin tubules.

Regardless of SiC paper grit used to grind dentin before cementing with RelyX Unicem, fracture morphology after shear bond strength testing was mainly adhesive between cement and dentin and combined fractures. When dentin was ground with P1200, some adhesive fractures between cement and ceramic were observed. The most frequent fracture morphology for Variolink Esthetic was also adhesive between cement and dentin, but a greater variation in fracture morphology was observed compared to RelyX Unicem (Figure 5). Figure 6 shows SEM images of the relation between dentin and the two cements.

## Discussion

In a previous publication by Sagen et al. [8] the tensile bond strength of ceramic rods cemented to dentin was studied. In the mouth, cemented restorations are exposed to forces in different directions, and to further investigate the performance of previously used surface treatments, shear bond strength was tested. The two test methods resulted in the same ranking of bond strength for the cements, implying that both methods are relevant. In addition, shear bond strength method is easier and faster [19].

The hypothesis that no difference in shear bond strength would be found for ceramics with different surface modifications was tested in the first part of the study. The hypothesis was accepted for four out of five cements (all but Multilink Automix) when comparing LDS to both zirconia groups, and for four out of five cements when comparing the zirconia groups to each other. Only Duo-Link showed a significantly higher bond strength for Zir E compared to Zir A. The difference might be related to the primer applied on the ceramic surface prior to cementation. When cementing zirconia rods using Duo-Link, Z-Prime Plus was applied on the surface. This primer exclusively enhances adhesion between resin cement and zirconia, alumina and metal substrates [20]. The superior effect of the primer on Zir E may be explained by a higher surface free energy for KHF_2_ etched compared to Al_2_O_3_ abraded zirconia as reported by Akazawa et al. [21]. Higher surface free energy increases the wettability of a surface [7,21]. The Sa-values and morphology of the two surfaces were also quite different as shown in Figure 1 and discussed in Sagen et al. [8]. For other cements a so-called ‘universal’ primer is recommended by many manufacturers [22,23]. These contain both functional monomers (i.e.10-Methacryloyloxydecyl dihydrogen phosphate) and silane [22], and might not be as effective in enhancing adhesion as specific zirconia primers.

LDS was used as reference material due to reliable adhesion to resin cement after HF etching and silane application [24]. This combination has long-term success rate [25]. In general, the results obtained for zirconia were comparable to those for LDS indicating that zirconia could successfully be cemented with resin cement.

The hypothesis of no effect of different resin cements on shear bond strength was rejected due to significantly higher bond strength promoted by some cements when looking at each ceramic separately. Variolink Esthetic resulted in bond strength in the highest range for all three ceramics, which might be related to the primer applied on the ceramic surface prior to cementation. Another explanation might be the cohesive strength of the cement, which is reflected in the low frequency of cohesive fractures after bond strength testing.

The five cements used in this study apply different adhesive mechanisms to bond to tooth substance. Duo-Link is a three-step etch-and-rinse adhesive, but even though such adhesives have been deemed optimal [14,26], this cement was found in the lower range of bond strength after both shear and tensile testing [8]. Variolink Esthetic is also an etch-and-rinse adhesive, but with only two steps. And even though the adhesive mechanism is similar, Variolink Esthetic performed significantly better in shear bond strength testing compared to Duo-Link. Two of the cements, Multilink Automix and Panavia F2.0, use a self-etching primer applied in one step. Panavia F2.0 resulted in significantly lower shear bond strength compared to all the other cements (Figure 2), as was also the case after tensile bond strength testing [8]. RelyX Unicem was the only self-adhesive cement tested, and the bond strength was similar to that of all other cements except Panavia F2.0. Based on results from fracture morphology characterization it seems that other factors than adhesive mechanism might have influenced the bond strength of ceramics cemented to dentin. One such factor is the content of inorganic filler particle, which affects viscosity and flowability of the cement [27].

In the second part of the study, a hypothesis of no effect of dentin surface roughness on shear bond strength for KHF_2_ etched zirconia cemented to dentin, was tested and partly rejected.

Variolink Esthetic and RelyX Unicem were selected for testing because of the highest frequency of adhesive fracture between cement and dentin (Figure 3), especially in combination with Zir E rods (Figure 3). This fracture type was also predominant in the second part of the bond strength study (Figure 5).

The SiC papers chosen corresponded to preparations burs with black (P80, very coarse) and yellow (P1200, extra-fine) color code [28,29]. The specific grits were selected to get an indication of whether a rough or smooth surface is clinically favorable for the bond strength of ceramic restorations. A wide variation in grit was considered necessary to evaluate this effect. The difference in roughness was confirmed by Sa-measurements performed in SEM. One would have expected a higher bond strength for a rougher surface, but this was not the case, and for Variolink Esthetic there was no difference in bond strength between the two surfaces (Figure 4). This indicates that other factors than the surface roughness affects the bond strength. The use of phosphoric acid etching to expose dentin tubules and assure penetration of adhesive and cement, seemed to be of importance [26,30,31]. Another factor could be viscosity of the cement [27]. Figure 6(a) shows resin tags in dentin tubules after shear bond strength testing of a ceramic rod cemented to dentin using Variolink Esthetic. The resin tags ensure a mechanical retention of the cement [30], which seems to be of more importance than the surface roughness for the cement bonded with an etch-and-rinse adhesive.

RelyX Unicem showed a significantly higher bond strength on smoother dentin surfaces (P1200) compared to on coarser dentin surfaces (P80). A smoother surface contributes to a thinner cement layer, which improves survival of the ceramic [32,33]. Remnants of RelyX Unicem on the dentin surface after shear bond strength testing was observed in SEM (Figure 6(b)). The remnants showed that the cement did not interfere with the tubules in dentin, but relied on chemical adherence and superficial interaction with dentin [34–36].

Shear bond strength testing of RelyX Unicem indicated that to attain the highest bond strength, dentin should be prepared with an extra-fine bur. For Variolink Esthetic, shear bond strength was not affected by the roughness of the dentin. An advantage when preparing with an extra-fine bur is a thinner cement layer compared to preparing with coarse burs [12]. This might affect the degree of microleakage [12] and survival of the restoration [32,33], and should be recommended regardless of the cement used. It has been shown that a cement layer thickness between 25 and 35 µm results in the highest tensile bond strength [18]. A thin cement layer (60 µm) also resulted in statistically significant higher bond strength compared to a thicker cement layer (180 µm) in a recently published study [37].

A limitation of the study was the high number of interfaces involving both dentin and ceramic. To study the effect of intervention on the different substrate surfaces, cementing ceramic to ceramic and dentin to dentin, respectively, could have been used [6,38].

Shear bond strength testing requires a plane surface to avoid error in the test conditions. Grinding of dentin using SiC paper on a horizontal grinding machine results in a more plane surface than what can be attained when preparing tooth substance in the mouth [39]. In the clinical situation both speed and direction of the dental bur varies. The clinical implication of the results might be affected by the grinding method and need to be addressed in a clinical study.

With the limitations of this study, it can be concluded that zirconia rods had similar bond strength to dentin as lithium disilicate glass-ceramic rods when using resin cement. A smooth dentin surface improved the bond strength of self-adhesive cement. Dentin surface roughness was not important for cement applied following an etch-and-rinse adhesive.
